# Scraps

**Published:** 1888-09

**Authors:** 


### SCRAPS

PICKED UP BY THE ASSISTANT-EDITOR.

---

**Holding On.**—The story comes from the West of a man so tenacious of lucre, that when he swallowed a five-dollar gold piece, the stomach-pump could only bring up $4.50.

**Truth through Error.**—It is said that a busy doctor sent in a certificate of death the other day, and accidentally signed his name in the space for "cause of death." The registrar says he wishes the profession would be as accurate generally.—*Journal of Reconstructives.*

**Counter-Irritation.**—A sad-looking man went into a drug-store and asked the druggist if he could give him something to drive from his mind thoughts of sorrow and bitter recollections. The druggist nodded, and put him up a mixture of quinine and wormwood and rhubarb and Epsom salts, with a dash of castor oil, and gave it to the man; and for six months he could not think of anything except new schemes for getting the taste out of his mouth.—*The Medical and Surgical Reporter.*

**"The Chief."**—Those who are acquainted with the history of antiseptic surgery, and with the not very remote traditions of the Edinburgh school, will have no difficulty in identifying the subject of the following lines taken from Mr. W. E. Henley's *A Book of Verses*, to which a subscriber has called my attention:

"His brow spreads large and placid, and his eye
Is deep and bright, with steady looks that still.
Soft lines of tranquil thought his face fulfill—
His face at once benign and proud and shy.
If envy scout, if ignorance deny,
His faultless patience, his unyielding will,
Beautiful gentleness, and splendid skill,
Innumerable gratitudes reply.
His wise, rare smile is sweet with certainties,
And seems in all his patients to compel
Such love and faith as failure cannot quell.
We hold him for another Herakles,
Battling with custom, prejudice, disease,
As once the son of Zeus with Death and Hell."

**"Plo Medinels."**—Some extraordinary specimens of "English as she is wrote" may be picked up at the Italian Exhibition at Earl's Court. Many of the exhibitors, with a view to recommending their wares to the British public, distribute amongst visitors circulars which pretend to be printed in our difficult language. The spelling and grammatical construction of most of these documents are open to hostile criticism; but, of all the circulars which we have succeeded in collecting, none contains more amusing faults than the pentaglot pamphlet which lauds the virtues of Dr. A. B.'s pills. We call the pamphlet a pentaglot inasmuch as it includes versions of the same statements in English, French, Spanish, German, and Italian. But its title-page is printed in Volapük, and is headed "Plo Medinels"—which, we suspect, means "For Medical Men." It should be equally appreciated by lay people.

The English section of this astonishing pamphlet begins with an extract from a paper—the translator calls it a note—which was read in 1887 before the Royal Lombard Institute of Science by Professor Joseph Sormani. "In this note," it is explained, "the eminent physiologist abridges the final results of his experiences made with the knowest antiseptics on the microbs of tubercolosys." After lightly touching on his experiments "made in gelatinous cultures, also against the tenaceous pathogen Koch's bacillus," the Professor is made to say: "at the rate of the easiness of its affording, of its solubility, and of its efficacy, the use of 'B.'s' pills is very recomended, at the dose of 4—10 pills in twenty-four hours." Dr. R. Guaita's translated testimony follows. "Therapeutics," says this authority, "must have the highest acknowledgment to A. B." The drug, "which is quickley decomposed by its very easy oxygenation, has been reduced by the inventor in form of pills covered with a thik sheet of resin, which, preveting the oxydation of the medicament, let the pill a long while unalterable. It is that the chief worth of this new pharmaceutical preparation." A pebble would have the same virtue. "The pills," continues Dr. Guaita, "melt easily in mouth, and have not disagreable taste, they are fortifying, make easy in extraordinary manner the expectoration, reduncig the muco-catarrhal secretion of the mucosa. Whence, their general use in the disease of the aerial tree, the larynx, the weasand, and the bronchiae." Dr. Guaita uses the pills "in the infantine Hospital directed by myself," especially in cases of "the retarded decision of pulmonite, the bronco-pulmonite, and generally in all the bronchio-pulmonary infirmities derived from the measles, the chin-cough," &c.; and has found that, after using them, "the breath was franker, the emphysema was sconjured, the tonicity generally returned to the large mucosa of the bronchio-pulmonary tree." Professor Tamburini, of Milan, says that the pills have given good results, even in "cases of lungs cavernity or cells." Professor Strambio thinks that "the renom" of the pills is merited. "The act of expectoration," he says, "is slight and long, and is not accompanied by feelings of loathing." The pills, he concludes, "must be carefully taken into consideration, and for that reason we warmly recommend them to all doctors." Dr. Luigi Casati, of Forli, is quite enthusiastic in his praises of the pills. "These pills are easily dissolved by the sole action of the saliva, and leaves a pleasant balmy taste which does not at all give disgust." We are told, incidentally, that the pills are used by "the director of the Clinical Poliambulance of Milano;" nor do we wonder at it, for Dr. Casati is hopeful "that they wil promptly sobstitute simple tar pearls, pastiles, pills, waters, and so many other preparations of any therapeutic valour." "Against a regular request made on a legal paper," the proprietors promise to send "a certain number of boxes" of the marvellous pills "for the opportune experiences." "The same firm, in order to publish shortly a large and particularized study on the terapeutical action" of their drug, "to which attend an eminent italian Physician, very well known for others important studies, beg ardently all the Physicians who experiments this new medicament, to communicate it the results of their tastes." The British public is, no doubt, anxious to know of a pill which, while reduncig the muco-catarrhal secretion, benefits the weasand, and, according to the knowest experts, fortifies the aerial tree in cases of pulmonite, lungs cavernity, and chin-cough; but a somewhat simpler statement of the virtues of Dr. B.'s medicine would have been welcome. We await with curiosity the publication of an English version of the large study to which attend an eminent italian Physician.—*St. James's Gazette.*

Dr. Von Mosetig-Monbrof, speaking at the Vienna Medical Congress, showed himself to be possessed of great originality in one thing—his bold and unique treatment of the English language. He quoted the following as Lister's statement of his "fundamental satze": (1) "Let the wound to be alone;" (2) "Let the wound to be protected;" (3) "Let to the wound her free discharge." This interesting specimen of "English as she is spoke" by Sir Joseph Lister will doubtless surprise many people; not least, we should imagine, Sir Joseph Lister himself.—*The Hospital.*

